# Implementing the “Common Skills Framework” in the Greek public sector: a Delphi approach

**DOI:** 10.3389/fsoc.2026.1866930

**Published:** 2026-06-19

**Authors:** Sofia K. Papageorgiou, Fotios Chatzitheodoridis, Konstantinos Spinthiropoulos, Stavros Kalogiannidis, Efstratios Loizou

**Affiliations:** 1 Department of Management Science and Technology, University of Western Macedonia, Kozani, Greece; 2 Department of Business Administration, University of Western Macedonia, Kozani, Greece

**Keywords:** Common Skills Framework (CSF), competencies, Delphi method, Greece, public administration, strategic human resource management (SHRM)

## Abstract

**Introduction:**

Competency frameworks are increasingly promoted as instruments for strengthening administrative capacity and modernizing public human resource management systems. However, their effectiveness depends not only on formal adoption but also on how they are interpreted, integrated, and enacted within everyday administrative practice. This study examines the implementation of the Common Skills Framework (CSF) in the Greek public sector, focusing on its contribution to professional development, HRM practices, and the organizational conditions shaping its integration.

**Methods:**

The study employs a modified e-Delphi methodology involving experts from key public-sector institutions, employee representatives, and academics. Two iterative rounds were conducted to evaluate consensus, stability, and divergence across core implementation dimensions related to competency framework adoption and organizational integration.

**Results:**

The findings indicate that the CSF is generally perceived as conceptually coherent and supportive of human resource management and professional development. Nevertheless, its practical implementation appears uneven and is strongly influenced by organizational culture, communication processes, managerial engagement, and administrative capacity. Participants emphasized that the successful integration of competency frameworks requires supportive institutional environments and continuous organizational learning mechanisms.

**Discussion:**

The study demonstrates that competency frameworks should not be viewed merely as formal policy instruments but rather as context-dependent and human-centered implementation processes. Their effectiveness is shaped by the interaction between organizational conditions, managerial support, and learning-oriented administrative cultures. The findings contribute to the literature on public sector reform by highlighting the conditions under which competency frameworks can support sustainable administrative capacity and long-term organizational development.

## 1 Introduction

In recent decades, public administrations have increasingly adopted competency frameworks as structured instruments for strengthening administrative capacity and improving human resource management (HRM) practices (Boyatzis, 2008; Armstrong and Taylor, 2014). As governance environments become more complex, digitalized, and performance-oriented, public-sector organizations are expected to align individual capabilities with strategic objectives in a systematic and transparent manner (Pollitt and Bouckaert, 2017; OECD, 2023). Within this broader reform trajectory, competency-based approaches have emerged as key mechanisms for clarifying role expectations, enhancing merit-based recruitment, and supporting continuous professional development (Skorková, 2016; Kruyen and Van Genugten, 2020).

Competency frameworks are generally understood as structured models that define the knowledge, skills, and behavioral attributes required for effective performance within specific organizational contexts (Boyatzis, 2008; Armstrong and Taylor, 2014). In public administration, they function as reference systems that connect individual capabilities with organizational objectives, supporting recruitment, evaluation, and development processes (OECD, 2017). Beyond their technical role, such frameworks reflect broader shifts toward capability-based management, emphasizing adaptability, learning, and alignment between individual performance and institutional goals (Tsolakidou, 2017; Kruyen and Van Genugten, 2020).

Despite their widespread adoption, the effectiveness of competency frameworks cannot be assumed based on formal institutionalization alone. Research in public management and human resource development consistently emphasizes that reform instruments are mediated by organizational context and implementation dynamics (Schein, 2010; Pollitt and Bouckaert, 2017). Competency models may function as meaningful developmental mechanisms, but they may also remain symbolic or procedural if not embedded within everyday administrative practice (Vidè et al., 2025). This highlights the need to examine not only whether such frameworks are adopted, but how they are interpreted, communicated, and operationalized within public organizations.

Recent literature points to several interrelated dimensions that shape these processes. Organizational culture and learning orientation influence the extent to which competency frameworks are integrated into daily work practices and linked to professional development (Guy and Mastracci, 2023). Communication and coordination processes affect how reform objectives are transmitted and understood across different administrative levels (OECD, 2017; Pollitt and Bouckaert, 2017). At the same time, managerial capacity plays a key role in translating competency models into operational practices and guiding their application within organizational settings (Armstrong and Taylor, 2014; Kruyen and Van Genugten, 2020). Together, these factors suggest that competency frameworks operate within complex socio-organizational environments, where their practical impact depends on more than their formal design.

From this perspective, increasing attention has been given to human-centred approaches to public sector HRM. Rather than viewing employees as passive recipients of reform instruments, this perspective emphasizes their role in interpreting, internalizing, and enacting organizational practices (Boyatzis, 2008; Armstrong and Taylor, 2014). The effectiveness of competency frameworks is therefore closely linked to employee engagement, perceived relevance, and opportunities for professional development. In this sense, competency-based reforms can be understood as processes that unfold within organizational contexts shaped by interaction, communication, and learning.

The Greek public administration introduced the Common Skills Framework (CSF) in 2022 under Law 4,940 as part of a broader effort to modernize HRM and strengthen administrative performance. The framework establishes a set of transversal competencies intended to guide recruitment, evaluation, and professional development across the public sector. In line with international and European developments in competency-based governance (OECD, 2017; European Commission, 2021), the CSF reflects an attempt to move toward a more integrated and capability-oriented model of human resource management.

However, despite its strategic significance, empirical evidence regarding the implementation of the CSF remains limited. There is a lack of research examining how the framework is perceived by practitioners, how it interacts with organizational conditions, and to what extent it supports professional development and HRM practices in practice. This gap is especially relevant given that the effectiveness of competency frameworks depends on their integration into everyday administrative routines rather than their formal adoption.

This study addresses this gap by examining the implementation of CSF through a human-centred perspective. Rather than focusing exclusively on its formal characteristics, the analysis explores how the framework contributes to professional development, supports HRM practices, and is shaped by organizational and managerial conditions. Drawing on a modified e-Delphi process involving experts from key public administration institutions, the study assesses patterns of consensus and divergence regarding the framework’s practical integration and developmental role.

In addition, the study contributes to the limited empirical literature on competency-based reforms in Southern European public administrations by combining an implementation-oriented perspective with a Delphi-based exploration of organizational and managerial dynamics. While existing research on competency frameworks has predominantly examined their formal design, structural characteristics, and integration into HRM systems (Skorková, 2016; OECD, 2017; Kruyen and Van Genugten, 2020), the present analysis emphasizes how such reforms are interpreted and operationalized within everyday administrative settings. Furthermore, the study moves beyond the descriptive mapping of competency frameworks by identifying implementation challenges and highlighting reform priorities emerging directly from stakeholder-informed expert assessments. In this sense, the analysis provides empirically grounded insights into the administrative dynamics that may facilitate or constrain the practical integration of competency-based reforms in the public sector.

By linking empirical findings with perspectives from public management and HRM literature, the article contributes to three main ways. First, it shifts the analytical focus from formal adoption to the practical enactment of competency frameworks within organizational settings. Second, it integrates a human-centred perspective into the analysis of public sector HRM reforms, emphasizing the role of employee engagement and professional development. Third, it provides empirically grounded insights into the conditions under which competency frameworks may function as developmental instruments in contemporary public administration systems.

The paper is structured as follows. The literature review section presents the conceptual framework, focusing on competency-based HRM and implementation dynamics. This is followed by the methodological approach, including the Delphi design and data analysis procedures. The results and discussion section presents empirical results across key thematic dimensions and interprets these findings in relation to the broader literature. The final section concludes with theoretical implications, practical recommendations, and directions for future research.

## 2 Literature review and conceptual framework

### 2.1 Strategic human resource management and competency-based governance

Over the past decades, public sector reforms have progressively shifted from traditional personnel administration models toward more integrated approaches associated with strategic human resource management (SHRM). This perspective emphasizes the alignment between workforce capabilities, organizational objectives, and broader governance priorities, recognizing human capital as a critical resource for achieving public sector effectiveness and responsiveness (Armstrong and Taylor, 2014; OECD, 2017).

Strategic human resource management places particular emphasis on the development and effective utilization of employees’ competencies within organizations. Rather than focusing primarily on administrative procedures related to staffing and employment regulation, SHRM highlights the role of human resource practices in supporting professional development, skill utilization, and employee engagement. In public sector organizations, this approach underscores the importance of creating conditions that enable civil servants to continuously develop their competencies, adapt to evolving professional demands, and contribute effectively to organizational performance (Armstrong and Taylor, 2014; OECD, 2017). Within this context, human resource management is expected to facilitate learning opportunities, support capability development, and encourage the meaningful use of employees’ knowledge and expertise in everyday administrative practice.

So, human resource management is not limited to regulating employment relationships but also plays a central role in fostering organizational learning and professional growth (Guy and Mastracci, 2023). Such approaches reflect a broader shift toward employee-centred management practices that view public employees not only as administrative personnel but as key contributors to institutional capacity and public value creation. Competency frameworks have emerged as key instruments through which strategic human resource management principles are translated into concrete human resource practices (Mavrommati et al., 2024; Chatzitheodoridis et al., 2022; Kalfas et al., 2023).

### 2.2 Competency frameworks in public sector human resource management

In the public administration and human resource management literature, skills are commonly associated with task-specific abilities, competencies are typically understood as broader, integrated combinations of knowledge, skills, and behavioral capacities that enable effective performance within specific organizational contexts (Boyatzis, 2008; Kruyen and Van Genugten, 2020; Armstrong and Taylor, 2014). Competency frameworks, therefore, extend beyond the identification of discrete skills by providing structured reference models that connect individual capabilities with institutional objectives and professional development pathways (OECD, 2017).

In the public sector, competencies function not merely as technical attributes but as context-embedded capacities shaped by organizational norms, governance expectations, and professional standards (Kruyen and Van Genugten, 2020). For analytical purposes, the Common Skills Framework (CSF)—although formally described as a “skills” framework—is approached as a competency-based HRM instrument. This interpretation does not redefine its legal status, but reflects the understanding that the framework articulates integrated behavioral and developmental expectations rather than isolated task abilities. The CSF is thus examined as a structured mechanism intended to support professional development and performance alignment within the Greek public administration, rather than as a static classification tool.

Competency frameworks have become central instruments in contemporary public sector human resource management (HRM), particularly within reform agendas aimed at enhancing meritocracy, transparency, and performance orientation (Armstrong and Taylor, 2014; OECD, 2017). In contrast to traditional personnel systems that prioritize formal qualifications and seniority, competency-based approaches emphasize integrated combinations of knowledge, skills, and behavioral capacities that enable effective performance within specific organizational contexts (Boyatzis, 2008; Skorková, 2016).

Within public administration, such frameworks are typically used to structure recruitment, performance appraisal, training, and career development systems (Kruyen and Van Genugten, 2020). From a strategic human resource management (SHRM) perspective, competencies function as mechanisms that align workforce capabilities with evolving governance demands, including digital transformation, collaborative policy environments, and citizen-centred service delivery (OECD, 2023). Rather than serving merely classificatory purposes, competency models are intended to guide behavioral expectations, clarify performance standards, and support targeted professional development.

International and European initiatives illustrate this broader shift. The OECD framework for a High-Performing Civil Service emphasizes leadership, analytical capacity, collaboration, integrity, adaptability, and digital literacy as core capabilities for modern public administrations, while progressively incorporating adaptability, digital literacy, and (OECD, 2017, 2022). Similarly, European competency initiatives seek to operationalize capability-based management through structured recruitment and interoperability-oriented skill models (European Commission, 2021). These frameworks reflect a shared understanding that administrative capacity increasingly depends on transferable and context-sensitive competencies rather than solely on procedural expertise. However, research consistently indicates that the presence of a formal competency framework does not automatically translate into substantive organizational change. The practical effectiveness of such instruments depends on their coherent integration into HRM systems and their acceptance within everyday administrative practice (Vidè et al., 2025). Competency frameworks may thus operate either as developmental mechanisms that foster skill utilization and professional growth, or as formal compliance tools with limited behavioral impact (Skordoulis et al., 2024; Kalogiannidis et al., 2022a, 2022b).

### 2.3 Organizational culture, learning orientation, and implementation dynamics

The implementation of competency frameworks is closely linked to organizational culture and learning orientation within public administrations. Organizational culture-understood as the shared assumptions, values, and norms shaping behavior within institutions (Schein, 2010)-influences how new management instruments are interpreted, internalized, and enacted. Reform tools may be formally adopted at the regulatory level, yet their practical meaning is negotiated within existing administrative routines, professional identities, and power structures (Pollitt and Bouckaert, 2017).

In public sector contexts characterized by rule-bound traditions and hierarchical authority, competency frameworks may be perceived either as developmental opportunities or as additional procedural requirements. Where compliance-oriented logics dominate, new HRM instruments risk being integrated superficially, without altering behavioral patterns or professional expectations. By contrast, learning-oriented cultures-those that encourage reflection, feedback, and continuous professional development-are more likely to support the substantive integration of competency models within everyday administrative practice (Guy and Mastracci, 2023). In such environments, competencies can function as shared developmental reference points rather than as evaluative checklists.

Communication and coordination processes also play an important role in shaping how competency frameworks are implemented within public administrations. Reforms in the public sector often involve multiple actors and institutional levels, including ministries, training institutions, recruitment authorities, and individual public organizations. As a result, the effective transmission of reform objectives and the consistent interpretation of competency frameworks depend on clear communication channels and coordinated institutional practices. When communication remains fragmented across hierarchical levels or between institutions, competency frameworks may be interpreted unevenly, limiting their practical influence on professional behavior and administrative routines (OECD, 2017; Pollitt and Bouckaert, 2017; Kalogiannidis et al., 2022a, 2022b).

Implementation dynamics further shape these outcomes. Public management reforms rarely unfold in linear or uniform ways; instead, they evolve through iterative processes influenced by managerial capacity, institutional readiness, and contextual constraints (Pollitt and Bouckaert, 2017). Managers and supervisors act as key mediators of reform, translating abstract competency language into operational expectations, development conversations, and performance practices (Pollitt and Bouckaert, 2017). Organizational leadership plays a decisive role in shaping how new HRM instruments are interpreted and embedded within institutional routines (Schein, 2010). Empirical research further suggests that implementation gaps in competency-based reforms often reflect differences in managerial capacity and organizational size rather than deficiencies in formal design (Vidè et al., 2025). Their interpretation, leadership, and engagement are therefore critical in determining whether competency frameworks become substantively embedded in everyday administrative practice.

From a human-centred perspective, competency frameworks contribute to administrative capacity when they clarify development pathways, support skill utilization, and foster professional empowerment (Boyatzis, 2008; Armstrong and Taylor, 2014). Public sector HRM literature emphasizes that competency-based approaches are most effective when linked to structured development systems and learning-oriented environments (Guy and Mastracci, 2023). Mechanisms such as mentoring, coaching, and experiential learning can reinforce this developmental orientation and enhance employee engagement and performance (Thaler et al., 2017; Vyas, 2019; Murray, 2003). Yet the institutionalization of such practices depends on organizational maturity and the availability of structured support systems (OECD, 2017). In the absence of supportive cultural, managerial, and communication conditions, competency frameworks may remain formally aligned with international standards while exerting limited influence on daily professional practice (Papaevangelou et al., 2023).

## 3 Methodology

### 3.1 Research design

In the context of public administration reform, the perspectives of practitioners, policymakers, and scholars are central to understanding how institutional frameworks are interpreted and operationalized in practice. In line with this orientation, it employs a Delphi approach to examine expert perceptions regarding the implementation of the Common Skills Framework (CSF). The Delphi method is a structured, iterative process designed to collect and refine informed judgments from a panel of experts through multiple rounds of controlled feedback (Dalkey and Helmer, 1963; Avella, 2016). It is particularly suitable for policy analysis in contexts where institutional instruments are relatively recent and empirical evidence remains limited (Avella, 2016; Barreteau, 2010).

The Delphi approach is structured around four core elements: anonymity of participants (Dalkey and Helmer, 1963; Avella, 2016); iteration through multiple rounds (Sekayi and Kennedy, 2017); controlled feedback (Dalkey and Helmer, 1963); and systematic aggregation and synthesis of responses (Avella, 2016). These elements ensure methodological rigor while fostering structured dialogue and the systematic collection of specialized knowledge within the expert panel.

Given that the CSF constitutes a recently introduced reform instrument within the Greek public administration, the Delphi approach was considered appropriate for capturing experiential knowledge, institutional insights, and evaluative perspectives from actors directly involved in its design and application.

### 3.2 Modified e-Delphi design and experts’ selection

Over time, several variations of the Delphi approach have emerged, including policy Delphi, real-time Delphi, and web-based or e-Delphi formats. These variations maintain the abovementioned core principles, while adapting the procedure to different research objectives and technological environments (Avella, 2016; Beiderbeck et al., 2021).

The present study adopts a modified e-Delphi approach, tailored to the requirements of public policy analysis and the practical constraints of expert participation. The process was conducted through a secure online environment (europa.eu platform), which ensured anonymity, facilitated participation from experts across different institutions, and enabled structured feedback between rounds. The digital format enhanced accessibility, temporal flexibility, and administrative efficiency, while preserving the methodological coherence of the classical Delphi design (Oliveira et al., 2025).

Participants were selected through a combination of experts from key institutions associated with the design, training, and implementation of the Common Skills Framework. The experts’ selection ensures both expertise and diversity of perspectives within the panel, incorporating policymakers, training specialists, recruitment authorities, experienced public managers, and academics specializing in public administration and human resource management (Giannarou and Zervas, 2014; Avella, 2016).

A total of 20 experts were invited to participate in the study. In the first round, 17 experts responded, including 6 policymakers, 3 employee representatives, 4 human resource management (HRM) professionals, and 4 academics. The panel consisted of 7 men and 10 women. In terms of educational background, 7 participants held doctoral degrees, 8 held master’s degrees, and 2 held bachelor’s degrees. The majority of participants (*n* = 14) occupied positions of responsibility or had specialization in HRM, while 6 reported direct involvement in the development of the CSF.

In the second round, 16 experts participated (6 men and 10 women), indicating a high retention rate between rounds. Among them, 5 held doctoral degrees, 9 held master’s degrees, and 2 held bachelor’s degrees. Similarly, most participants (*n* = 13) occupied positions of responsibility or had HRM-related expertise, while 4 reported involvement in the CSF development process.

Overall, the composition of the panel reflects a balanced representation of policy-level perspectives, administrative experience, and academic and HRM expertise, contributing to the robustness and analytical relevance of the Delphi findings.

### 3.3 Research questions and instruments

The questionnaire is used as the core analytical tool through which expert judgments are systematically collected, structured, and compared across successive rounds of consultation (Keeney et al., 2011; Avella, 2016). The questionnaire included both closed-ended and open-ended questions designed to capture expert assessments regarding the development, implementation, and potential impact of the Common Skills Framework (CSF) in the Greek public administration.

Especially for the present study, the analysis focuses exclusively on the closed-ended items of the questionnaire, which allow the examination of patterns of agreement and convergence through quantitative indicators such as median values and measures of dispersion. These items were formulated as evaluative statements using a five-point Likert scale ranging from strong disagreement to strong agreement. The statements were designed to capture key analytical dimensions related to the CSF’s implementation process, its alignment with professional development objectives, the organizational conditions influencing its application, and its perceived contribution to human resource management practices.

The study was conducted in accordance with established ethical standards for research involving human participants. The research instrument was approved by the Ethics and Deontology Committee of the University of Western Macedonia. Participation was voluntary, anonymity was ensured throughout the process, and informed consent was obtained from all participants (Keeney et al., 2011). Table 1 presents the analytical dimensions and corresponding closed-ended items (Q1–Q17) included in the Delphi instrument, providing a structured overview of the key thematic areas examined in the study.

**Table 1:** Analytical dimensions and closed-ended items included in the Delphi questionnaire (Q1–Q17).

Analytical dimension	Item	Statement
Perceptions of implementation	Q1	Stakeholders and participating actors adequately covered the key domains relevant to the development of the Common Skills Framework.
	Q2	Participation and consultation with relevant stakeholders contributed effectively to the development of the CSF.
	Q3	The development of the CSF was accompanied by clear monitoring and evaluation mechanisms.
Alignment with professional development objectives	Q4	The establishment of the CSF responded to real needs of the public administration.
	Q5	The CSF skills contribute to improving the effectiveness of public administration.
	Q6	The skills defined in the CSF align with the modernization needs of the Greek public administration.
	Q7	The skills defined in the CSF are clearly formulated and practically applicable.
	Q8	The CSF skills correspond to the personal and professional expectations of public employees.
	Q9	Some skills within the CSF have not been sufficiently highlighted.
	Q10	Some important skills are missing from the CSF and should be included.
Organizational and managerial conditions influencing implementation	Q14	Public employees experienced the introduction of the CSF as a positive change.
	Q15	Public employees experienced the introduction of the CSF as part of ongoing legislative adjustments in the evaluation system.
Innovative skill development practices	Q11	Coaching improves the professional and personal skills of public employees.
	Q12	Mentoring improves the professional and personal skills of public employees.
	Q13	On-the-job training improves the practical skills of public employees.
Contribution to HRM practices	Q16	The CSF supports improvements in HRM practices in the public sector.
	Q17	The CSF contributes to professional development and better utilization of employees’ skills.

### 3.4 Procedure and rounds

The study was developed with the implementation of the Delphi process in two structured rounds of expert consultation, consistent with the multi-round logic of the Delphi approach (Dalkey and Helmer, 1963; Sekayi and Kennedy, 2017). The first round focused on collecting expert assessments through a combination of closed-ended (Likert-scale) and open-ended questions, allowing both quantitative comparison and qualitative exploration of expert perceptions regarding the development and implementation of the Common Skills Framework (CSF).

Following the completion of the first round, responses were aggregated and analyzed using descriptive statistical indicators, including measures of central tendency and dispersion. A summary of the results was then provided to participants as controlled feedback, enabling them to review the overall distribution of responses and reflect on their initial evaluations.

**Figure 1:**
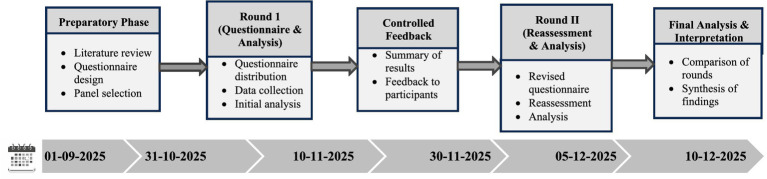
Delphi process and research timeline.

The second round invited participants to reassess selected issues and prioritize key aspects of the framework’s implementation based on the feedback from the first round. This stage aimed to refine expert judgments and identify areas of emerging convergence or persistent divergence among participants. The two-round structure is widely regarded as sufficient in applied Delphi studies where the objective is to identify patterns of convergence and support structured expert reflection rather than achieve full unanimity (Avella, 2016). Figure 1 illustrates the overall structure of the Delphi process followed in this study, including the two iterative rounds and the role of controlled feedback in supporting the refinement of expert judgments.

The data generated through this iterative process were subsequently analyzed using quantitative techniques, as described in the following section.

### 3.5 Data analysis

In line with Delphi methodology, a reduction in response variability across rounds was interpreted as an indication of increasing convergence among expert judgments (Dalkey and Helmer, 1963; Keeney et al., 2011; Giannarou and Zervas, 2014). Mean, median, standard deviation (SD), coefficient of variation (CV), and interquartile range (IQR) were employed to assess central tendency and response dispersion, a common analytical approach in Delphi-based consensus research (Giannarou and Zervas, 2014). Given that the questionnaire items were measured using a five-point Likert scale, responses were treated as ordinal data, and median values were used as the primary indicator of central tendency in the interpretation of expert judgments.

**Figure 2:**
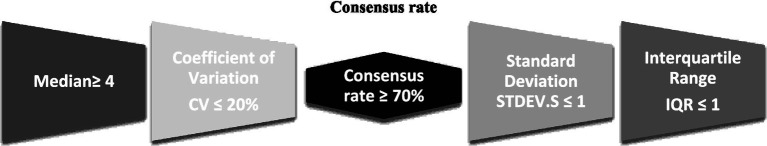
Statistical criteria used to establish consensus in the present study. Source: Author’s adaptation based on Mintzberg (1998), Giannarou and Zervas (2014), Sarbah and Otu-Nyarko (2014), and Tsakiridou et al. (2022).

Consensus among expert responses was evaluated using a combination of statistical and proportional criteria. As illustrated in Figure 2, convergence was assessed through indicators of central tendency and variability together with a proportional agreement threshold.

In accordance with established Delphi guidelines (Giannarou and Zervas, 2014), consensus was considered achieved when at least two statistical criteria were satisfied in combination with a minimum agreement rate of 70%. This approach integrates indicators of central tendency, variability, and proportional agreement to provide a structured assessment of response alignment across the expert panel.

## 4 Results and discussion

Table 2 synthesizes a comparative overview of expert assessments across the two Delphi rounds (Q1–Q17), including median and standard deviation values. The table highlights patterns of stability and convergence, as well as items that were not re-assessed in the second round.

**Table 2:** Comparison of expert assessments across Delphi rounds (Q1–Q17).

Item	Round I Md	Round I SD	Round II Md	Round II SD	Notes
Q1	3	0.88	3	0.72	Retained
Q2	3	1.05	–	–	Not included in Round II
Q3	3	0.99	3	0.86	Retained
Q4	4	1.00	4	1.00	Retained
Q5	4	1.23	–	–	Consensus achieved in Round I
Q6	4	1.09	4	0.91	Retained
Q7	3	0.97	4	1.03	Retained
Q8	3	1.01	–	–	Not included in Round II
Q9	4	0.68	–	–	Consensus achieved in Round I
Q10	4	0.88	4	0.58	Retained
Q11	2	1.06	–	–	Not included in Round II
Q12	2	1.22	2	0.91	Retained
Q13	3	1.01	–	–	Not included in Round II
Q14	3	0.97	3	0.70	Retained
Q15	4	0.87	–	–	Consensus achieved in Round I
Q16	4	0.86	–	–	Consensus achieved in Round I
Q17	4	1.23	–	–	Consensus achieved in Round I

**Figure 3:**
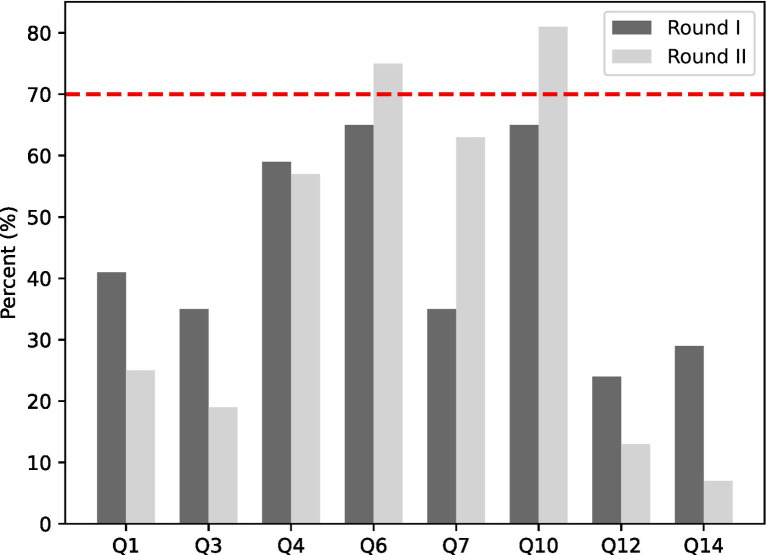
Change in agreement levels between Delphi rounds (percentage of responses “Agree” and “Strongly agree”).

Across the two-round e-Delphi process, experts’ perceptions of the Common Skills Framework (CSF) reveal a pattern of moderate agreement regarding the overall implementation trajectory, combined with persistent variability on process-oriented aspects of adoption. Figure 3 presents the distribution of agreement levels across core items between the two Delphi rounds. To facilitate interpretation, a horizontal dashed line is included in the figure to indicate the 70% consensus threshold.

The visualization confirms that while several items remain centred around neutral or moderate agreement levels, some items demonstrate increasing concentration of responses between rounds. In particular, items related to the strategic relevance of the CSF (Q4—“The establishment of the CSF responded to real needs of the public administration” and Q6—“The skills defined in the CSF align with the modernization needs of the Greek public administration”) exhibit higher agreement levels compared to questions addressing the procedural aspects of implementation.

In Round I, items addressing the inclusiveness of the adoption process were positioned around the neutral point (Q1—“Stakeholders and participating actors adequately covered the key domains relevant to the development of the CSF”: Md = 3), accompanied by moderate dispersion in expert responses. In Round II, response variability decreased while the median remained unchanged, suggesting a gradual stabilization of expert judgments rather than a substantive shift in their overall assessment.

A similar pattern is observed in items capturing governance and monitoring arrangements. The statment Q3—“The development of the CSF was accompanied by clear monitoring and evaluation mechanisms” remained centred at Md = 3 in both rounds, with only marginal improvements in variability (SD = 0.99 → 0.86; CV = 31.84% → 31.14%; IQR = 1 → 1). This suggests that, although controlled feedback may have supported a gradual increase in agreement, the expert panel continued to view implementation as unevenly structured, particularly in terms of systematic follow-up and evaluation.

Perceptions regarding the alignment of the Common Skills Framework (CSF) with professional development objectives are comparatively strong, particularly in items that connect the framework with modernization and administrative effectiveness. Q4—“The establishment of the CSF responded to real needs of the public administration” is consistently positioned in the agreement range (Md = 4 in both rounds), indicating that experts broadly recognize the framework as addressing substantive institutional needs. Similarly, Q5 and Q6 suggest that the CSF is perceived as contributing to administrative effectiveness and aligning with broader modernization priorities.

At the same time, items related to the clarity and practical applicability of the framework reveal more moderate assessments. Statement Q7—“The skills defined in the CSF are clearly formulated and practically applicable” is centered around the neutral range, suggesting that while the framework is considered relevant at a strategic level, its operational clarity may be less consistently perceived across different administrative contexts. A comparable pattern emerges in items capturing the relationship between the CSF and employees’ professional expectations. Q8—“The CSF skills correspond to the personal and professional expectations of public employees” remains in the mid-range, indicating that the alignment between formal competency definitions and individual-level expectations is not fully consolidated. Further complexity is reflected in items addressing potential gaps within the framework. Q9 and Q10 indicate that experts identify areas where certain skills are either insufficiently emphasized or entirely absent. This suggests that, despite its overall relevance, the CSF is perceived as an evolving instrument that may require further refinement in order to fully support competency development within the public sector.

Taken together, these findings suggest that CSF is broadly understood as a development-oriented HRM instrument aligned with modernization priorities. However, the variation in responses across items related to clarity, applicability, and skill coverage indicates that this alignment is not yet fully translated into a coherent and consistently operationalized framework. This highlights the importance of continuous adaptation and contextualization in the implementation of competency-based HRM systems.

This result is consistent with approaches to adult learning in the public sector, which emphasize that competency frameworks become meaningful only when supported by structured learning processes and continuous professional development opportunities. In this sense, the effectiveness of the CSF depends not only on its formal design but also on the extent to which it is linked to training activities that facilitate the practical development of employees’ skills (Komseli, 2023; Komseli and Bourbouli, 2022).

**Figure 4:**
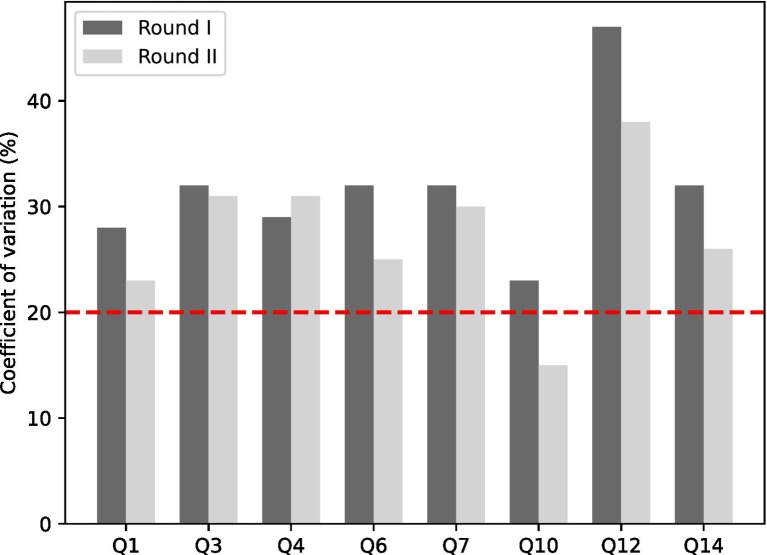
Evolution of response dispersion between Delphi rounds (SD, CV, and IQR indicators).

Figure 4 presents the evolution of response dispersion using standard deviation (SD), coefficient of variation (CV), and interquartile range (IQR) indicators. To enhance interpretability, a horizontal dashed line is included to indicate the threshold for strong convergence (CV ≤ 20%).

The reduction in dispersion observed in several items suggests a gradual convergence of expert assessments following the controlled feedback process. Rather than reflecting substantial shifts in central tendency, the Delphi process appears to have contributed primarily to stabilizing expert judgments regarding the conditions necessary for effective implementation. This pattern indicates that experts increasingly converge on a shared understanding that implementation depends on enabling organizational conditions.

At the same time, the persistence of variability in selected items points to the influence of contextual and organizational factors that shape how the CSF is interpreted and applied in practice. Differences in communication structures, levels of managerial engagement, and the presence (or absence) of learning-oriented environments appear to contribute to uneven patterns of implementation across organizational settings. This interpretation is further supported by the observed divergence in areas related to learning and development practices, suggesting that the effectiveness of competency-based frameworks depends not only on formal institutional design but also on the existence of supportive organizational infrastructures and managerial mediation mechanisms.

Overall, the findings highlight that competency frameworks operate as context-dependent HRM instruments, whose practical impact is shaped by organizational readiness, communication dynamics, and leadership capacity. As a result, their implementation should be understood as a socially mediated process rather than a purely technical or regulatory intervention.

This pattern is also consistent with approaches that emphasize the role of managers as facilitators of learning and communication processes within public organizations. In this perspective, managers do not merely implement formal HRM tools but contribute to shaping how competency frameworks are interpreted, communicated, and applied in everyday administrative practice (Bourbouli, 2020).

The perceptions of the applicability and effectiveness of innovative skill development practices—coaching, mentoring, and on-the-job training—within the Greek public sector were marked by substantial divergence. Coaching (Q11—“Coaching improves the professional and personal skills of public employees”) received comparatively low evaluations in Round I and exhibited substantial variability in expert responses. Due to the absence of convergence among participants, the item was excluded from subsequent Delphi rounds. On-the-job training (Q13—“On-the-job training improves the professional skills of public employees”) similarly showed high variability (Md = 3; SD = 1.01; CV = 35.93%; IQR = 2) and was therefore excluded from Round II. Mentoring (Q12—“Mentoring improves the professional and personal skills of public employees”) was reintroduced and showed a modest statistical improvement in Round II (dispersion reduced from SD = 1.22; CV = 46.15% to SD = 0.91; CV = 37.93%), but the median remained low (Md = 2), and variability remained above typical consensus thresholds used in Delphi designs. This indicates that, even after feedback, mentoring is not perceived as a widely institutionalized or consistently effective practice across the public sector.

The findings do not necessarily imply rejection of innovative developmental practices, but they suggest that such mechanisms remain unevenly institutionalized and may depend heavily on managerial initiative, local organizational culture, and the availability of supportive learning infrastructures. This pattern is consistent with research emphasizing that competency-based HRM systems require learning-oriented organizational environments to translate formal capability models into effective professional development practices.

In Round I, experts reported agreement that the CSF supports improvements in HRM practices (Q16—“The CSF supports improvements in HRM practices in the public sector”: Md = 4; SD = 0.86; CV = 23.63%). A comparable pattern emerges in perceptions related to professional development and the utilization of employees’ skills (Q17—“The CSF contributes to professional development and better utilization of employees’ skills”: Md = 4; SD = 1.23; CV = 34.22%). In substantive terms, Q17 is particularly informative, as it captures a shared recognition that a structured competency framework can clarify expectations and serve as a developmental reference point for employees. This interpretation aligns with strategic human resource management approaches that emphasize capability development, skill alignment, and employee-centred professional growth within public organizations.

Overall, the Delphi results indicate that the CSF is broadly perceived as supporting improvements in public-sector HRM, particularly as a mechanism that can structure recruitment, evaluation, and professional development practices beyond a purely classificatory role. However, the extent to which these perceived benefits are realized in practice appears to depend on organizational and managerial conditions, as discussed in the previous subsections.

**Figure 5:**
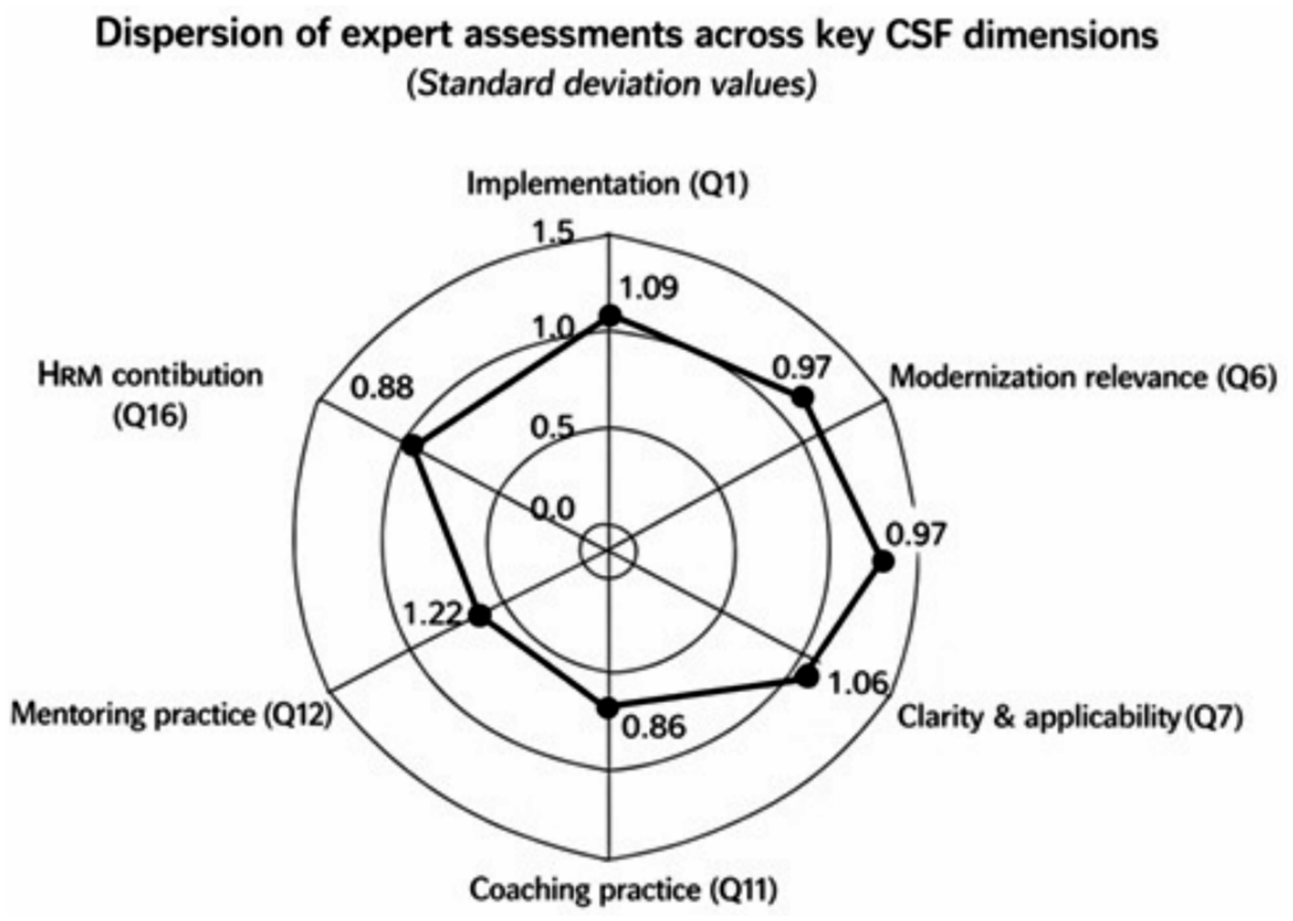
Dispersion of expert assessments across key CSF-related dimensions. The radar chart illustrates variability in expert responses across selected dimensions related to implementation, learning practices, employee perceptions, and HRM contribution.

To provide a complementary perspective on expert assessments, Figure 5 visualizes the dispersion of responses across selected CSF-related dimensions using standard deviation values. The figure highlights differences in the degree of variability among expert judgments, revealing areas where perceptions are more heterogeneous.

The radar chart shows that higher levels of dispersion are observed in items related to innovative learning practices (e.g., coaching and mentoring), indicating substantial divergence in expert views regarding their applicability and effectiveness within the Greek public administration. In contrast, lower variability is observed in items associated with the CSF’s contribution to HRM practices (e.g., Q16), suggesting a higher degree of convergence in expert assessments.

The results highlight that while the CSF is consistently perceived as a relevant HRM instrument, greater uncertainty and divergence emerge in relation to its practical implementation and associated development practices. These findings reinforce the interpretation that the effectiveness of competency-based reforms depends not only on their formal design but also on organizational context, managerial capacity, and the availability of learning-oriented support mechanisms.

### 4.1 Analysis and critical assessments of findings

Even if the Common Skills Framework (CSF) is broadly perceived as a relevant and strategically aligned reform initiative, its practical implementation appears uneven and contingent upon organizational and managerial conditions in Greece. In particular, the results highlight the centrality of employee-centred dynamics, learning-oriented environments, and managerial mediation, while also revealing persistent limitations in communication processes, both within organizations and across administrative units. These findings suggest that the effectiveness of competency frameworks depends not only on their formal design but also on the extent to which they are embedded in everyday administrative practice through communication, leadership, and professional development mechanisms (Armstrong and Taylor, 2014; Pollitt and Bouckaert, 2017; Terzi et al., 2017; Tsolakidou, 2017; Kruyen and Van Genugten, 2020).

A first key insight concerns the limited extent to which the implementation of the CSF is experienced as an employee-centred process. Although the framework is generally recognized as relevant to modernization and professional development objectives, the findings indicate that its translation into a positive employee experience remains moderate and uneven. This suggests that competency frameworks do not automatically generate engagement or psychological ownership among employees, even when they are normatively aligned with organizational goals. In line with human resource management literature, the effectiveness of such instruments depends on the extent to which employees understand, internalize, and actively engage with the competencies defined (Boyatzis, 2008; Armstrong and Taylor, 2014). The relatively moderate evaluations regarding employee experience point to a gap between formal alignment and lived organizational reality, indicating that greater emphasis is needed on participation, feedback, and meaningful integration into daily work practices. This interpretation is also consistent with human-centred approaches to public administration, which emphasize the role of employee engagement and skill development in supporting organizational effectiveness (Bourbouli, 2023).

Closely related to this is the role of organizational learning culture. The findings reveal that while the CSF is conceptually linked to professional development, the supporting learning environment appears underdeveloped or unevenly institutionalized. The divergence observed in perceptions regarding coaching, mentoring, and on-the-job training suggests that such practices are not consistently embedded across the public sector. This aligns with existing research emphasizing that competency-based HRM systems require learning-oriented organizational contexts in order to translate formal capability models into effective professional development mechanisms (Guy and Mastracci, 2023). In parallel, research on adult learning in the public sector highlights that competency development depends on continuous training, structured educational interventions, and alignment between learning processes and organizational needs (Komseli, 2023; Komseli and Bourbouli, 2022). In the absence of such conditions, competency frameworks risk remaining declarative tools rather than drivers of continuous skill development.

A further critical dimension emerging from the findings is the role of managerial mediation and communication processes in shaping implementation outcomes. This is reflected in items related to stakeholder participation (Q1–Q2) and monitoring mechanisms (Q3), as well as in the moderate evaluation of employees’ experience (Q14), suggesting that communication and coordination remain unevenly developed. These patterns indicate that the CSF has not been consistently communicated or internalized across organizational levels.

This interpretation aligns with research emphasizing that public sector reforms are mediated through organizational actors and communication processes rather than determined solely by formal design (Pollitt and Bouckaert, 2017). From a strategic human resource management perspective, effective implementation of competency frameworks depends on managerial capacity to translate abstract competency models into meaningful guidance and everyday practice (Armstrong and Taylor, 2014). Moreover, research on competency-based HRM highlights that competencies are context-dependent and require active interpretation and communication within organizational settings (Kruyen and Van Genugten, 2020). In this sense, managers act as key intermediaries who can either facilitate or constrain the effective embedding of competency frameworks, depending on their capacity to support communication and link competencies with professional practice. This view is reinforced by approaches that conceptualize managers as facilitators of learning and communication processes within organizations, acting as “coaches” who support the practical application of competencies (Bourbouli, 2020).

These dynamics are closely connected to a broader structural gap between formal design and everyday administrative practice. The results consistently indicate that while the CSF is positively assessed at the level of strategic intent, its operationalization remains uneven and context dependent. This supports the argument that public sector reforms often exhibit a decoupling between formal adoption and practical implementation (Pollitt and Bouckaert, 2017). Competency frameworks may therefore function as formal compliance instruments unless they are supported by appropriate organizational conditions, including managerial engagement, communication infrastructures, and learning-oriented cultures. The persistence of moderate agreement and non-trivial dispersion across several items reflects this tension between formal alignment and practical variability.

At the same time, the findings point to the potential of the CSF as a developmental human resource management instrument. Experts broadly recognize its contribution to improving HRM practices and supporting the professional development and better utilization of employees’ skills. This suggests that competency frameworks can provide a common reference point for structuring recruitment, evaluation, and development processes, in line with strategic HRM approaches that emphasize capability development and alignment (Armstrong and Taylor, 2014). This interpretation is also supported by research highlighting the strategic role of competency frameworks in integrating HRM functions and supporting organizational development (Tsolakidou, 2017). However, this potential appears conditional upon the presence of enabling organizational factors. Without effective communication, managerial support, and learning infrastructures, the developmental dimension of the CSF is unlikely to be fully realized.

## 5 Conclusion

This study examined the implementation of the Common Skills Framework (CSF) in the Greek public administration through a Delphi-based analysis of expert perceptions, focusing on its role as a competency-based human resource management instrument. The findings indicate that while the CSF is broadly perceived as a relevant and strategically aligned reform, its practical impact remains contingent upon organizational and managerial conditions. In particular, the results highlight the importance of employee engagement, communication processes, managerial capacity, and learning-oriented environments in shaping the extent to which competency frameworks are effectively embedded in everyday administrative practice.

From a theoretical perspective, the study contributes to the literature on competency-based HRM and public sector reform by emphasizing the importance of implementation dynamics and organizational context. Rather than treating competency frameworks as neutral or technical tools, the findings underscore their socio-organizational character, showing that their effectiveness depends on processes of interpretation, communication, and integration within specific institutional settings. In doing so, the study supports and extends existing research on the gap between formal reform design and practical implementation, highlighting the role of employee-centred and context-sensitive approaches in understanding public sector HRM transformations.

In terms of practical implications, the findings suggest that the successful implementation of competency frameworks requires more than formal adoption. Public organizations should invest in strengthening communication mechanisms, both within and across administrative units, in order to ensure that competency frameworks are clearly understood and consistently applied. In addition, greater emphasis should be placed on managerial development, enabling supervisors to effectively translate competency models into everyday practice through guidance, feedback, and performance management processes. The development of structured learning environments—through mentoring, coaching, and experiential learning—also emerges as a critical factor for supporting the practical utilization of competencies and enhancing employee engagement.

This distinction is particularly important in the context of public administration reforms, where formal policy adoption may not necessarily correspond to uniform implementation practices across organizations. Consequently, the findings should be interpreted as analytically informed assessments of implementation dynamics rather than direct measurements of employees’ day-to-day experiences or organizational behavior.

The study is subject to certain limitations. The Delphi method captures expert perceptions rather than direct organizational behavior, and the findings therefore reflect informed assessments rather than observed practices. Moreover, expert assessments may not fully capture the variability of implementation practices across different public organizations and administrative levels. Perceptions regarding the effectiveness, applicability, or organizational integration of the CSF may differ from the ways in which the framework is enacted in everyday administrative routines. Consequently, future research could complement Delphi-based expert evaluations with organizational case studies, employee-level evidence, or longitudinal implementation analyses in order to examine how competency-based reforms operate in practice across diverse institutional settings.

In addition, the focus on the Greek public administration limits the generalizability of the results to other administrative contexts. Furthermore, the analysis is based on a two-round Delphi process, which, while sufficient for identifying patterns of convergence, may not fully capture the evolution of expert consensus over time.

Although the findings emerge from the Greek public administration, several of the identified dynamics—such as the importance of managerial mediation, communication processes, and learning-oriented organizational cultures—may also be relevant to other public administration systems undergoing competency-based modernization reforms. Nevertheless, differences in administrative traditions, institutional capacity, and reform trajectories may influence the extent to which these findings are transferable across national contexts.

Future research could build on these findings by examining the implementation of competency frameworks through qualitative case studies or mixed method approaches that explore how competencies are enacted in everyday administrative settings. Comparative studies across different national contexts would also be valuable in identifying how institutional and cultural factors influence the effectiveness of competency-based HRM systems. Finally, future research could complement expert-based evidence with quantitative studies at the employee level, investigating how communication, participation, and learning opportunities shape the internalization, utilization, and perceived impact of competency frameworks in the public sector.

The findings of the study further suggest that future research should move beyond the formal design of competency frameworks and examine how such reforms are implemented within different organizational environments and administrative cultures. In particular, the observed divergence regarding innovative learning practices and the importance of managerial and organizational conditions indicate the need for comparative studies exploring how competencies are identified, interpreted, and operationalized across different public-sector contexts.

## Data Availability

The raw data supporting the conclusions of this article will be made available by the authors, without undue reservation.
